# “Be perfect in every respect”: the mediating role of career adaptability in the relationship between perfectionism and career decision-making difficulties of college students

**DOI:** 10.1186/s40359-022-00845-1

**Published:** 2022-05-27

**Authors:** Huaruo Chen, Liman Pang, Fei Liu, Tingting Fang, Ya Wen

**Affiliations:** 1 Institute of Vocational and Adult Education, East China Normal University, Shanghai, 200062 China; 2 Center for Research and Reform in Education, Johns Hopkins University, Baltimore, 21286 USA; 3 School of Education Science, Nanjing Normal University, Nanjing, 210046 China; 4 School of Psychology, Huaiyin Normal University, Huaian, 223300 China; 5 School of Psychology, Naning Normal University, Nanjing, 210046 China; 6 School of Teacher Education, NanJing XiaoZhuangl University, Nanjing, China

**Keywords:** Perfectionism, Career adaptability, Career decision-making difficulties, College students

## Abstract

**Background:**

Considering the weakening of the economy and the shrinkage of jobs under the current global epidemic crisis, the employment of college graduates is facing unprecedented and cruel competition. However, many college students lack a reasonable understanding and orientation of themselves, which leads to them having high expectations for future careers and not considering whether they are competent or not. Due to a lack of ability and self-confidence, they appear to be at a loss and hesitant when facing career choices. Therefore, the purpose of this study is to explore the relationship between perfectionism, career adaptability and career decision-making difficulties from both positive and negative aspects.

**Methods:**

We sampled 400 college students in China and used a questionnaire to obtain cross-sectional data through the Perfectionism, Career Adaptability, and Career Decision Difficulties scales. This study explored the relationship between them using statistical analysis software such as SPSS and AMOS through the results of correlation analysis and mediating role analysis.

**Results:**

College students’ perfectionism, career adaptability, and career decision-making difficulties were significantly correlated (*p* < 0.01). Positive perfectionism has a negative predictive effect on career decision-making difficulties, and career adaptability plays a completely mediating role in it (the direct effect is −0.032, the mediation effect is −0.244, and the total effect is −0.276.). Negative perfectionism plays a positive predictive role in career decision-making difficulties, and career adaptability plays a part in mediating roles (the direct effect is 0.334, the mediating effect is 0.099, and the total effect is 0.433.).

**Conclusions:**

College students will more or less pursue “perfect”, but often with their own preferences to pursue, cannot be done based on the rational analysis of things to pursue perfect. College students have higher positive perfectionism and negative perfectionism, and their career adaptability is also at a higher level, but there is a higher degree of career decision-making difficulties. Positive perfectionism of college students can reduce the difficulty of career decision-making, and career adaptability plays a completely mediating role in it. Negative perfectionism of college students will lead to difficulties in career decision-making, in which career adaptability plays a mediating role.

## Introduction

Faced with the economic impact of the epidemic and the shortage of talent in the labor market, the increasing number of graduates has led to the reduction of job opportunities for college students [1]. The mismatch between the students trained by the school and the talents demanded by the employing unit aggravates the employment difficulties for graduates [2]. The employment problem of college graduates not only attracts the close attention of students and parents but also becomes the focus of the attention of schools and society [3]. Faced with the serious employment situation, the phenomenon of college students’ difficulties in choosing a job and various negative emotions have made colleges and universities pay increasing attention to psychological and career counseling for college students [4]. Some scholars believe that the difficulty in career decision-making of college students is due to the high expectations of their careers [5]. Perfectionism is thought to have two sides. One view is that people see it as a negative personality trait [6], perfectionism would set a higher goal in order to meet their own or the expectations of others around, which always bring fear failure, and hesitation when they made career decisions. This is called maladaptive perfectionism [7]. Some researchers also mention perfectionism as a negative attitude of graduates in the employment counseling process [8]. However, another view holds that the pursuit of excellence and perfection is the key to their success [9]. This is called adaptive good perfectionism [10]. Among them, adaptive good perfectionism refers to self-perfectionism and tends to perfectionism’s active pursuit of high standards. Maladaptive perfectionism is socially determined perfectionism, which means that perfectionists feel important to others and demand high standards from themselves. In this way, it seems that the influence of perfectionism on career decision-making difficulties can be explored from both positive and negative aspects.

In addition, college students’ career adaptability has become a problem that people gradually pay attention to [11]. Resilience is an emerging variable in the field of career development and core competency in addressing career challenges posed by the rapidly changing realities of society [12]. Individuals with high career adaptability can adapt to changes in career roles more quickly and better cope with the difficulties and unexpected situations encountered at work [13]. Germijs and Verschueren (2007) show that young people’s career adaptability has a significant impact on the quality of planning, exploration, confidence, and decision-making during their career transitions [14]. Career decisions are easier to make if you have reasonable planning, positive exploration, and therefore strong confidence in career transition. It seems that career adaptability can promote the improvement of career decision-making ability and reduce the occurrence of career decision-making difficulties. In addition, career adaptability is also affected by individual and other antecedent variables, such as openness, preciseness, extroversion, and affinity [15]. On the one hand, positive perfectionists tend to set flexible goals according to their own characteristics, such as being able to actively pursue organizational rationality and maintaining an optimistic attitude towards the results [16]. On the other hand, negative perfectionists tend to set rigid goals and fear failure and are prone to anxiety [16]. It can be deduced that positive perfectionism can promote career adaptability, while negative perfectionism has the opposite effect. Therefore, career adaptability is included in the present study as an intermediary variable in the process of perfectionism affecting career decision-making difficulties.

### Purpose of the study

The purpose of this study is to explore the relationship between perfectionism, career adaptability and career decision-making difficulties from both positive and negative aspects. On the one hand, it explores the predictive role of positive perfectionism on college students’ career decision-making difficulties and tests the intermediary role of career adaptability. On the other hand, it explores the predictive role of negative perfectionism on career decision-making difficulties and tests the intermediary role of career adaptability. By exploring the psychological mechanism behind college students’ career decision-making difficulties, the personality trait of perfectionism influences college students’ career decision-making through career adaptability in both positive and negative aspects, thus providing help for the intervention of decision-making difficulties.

## Literature review

**Fig. 1:**
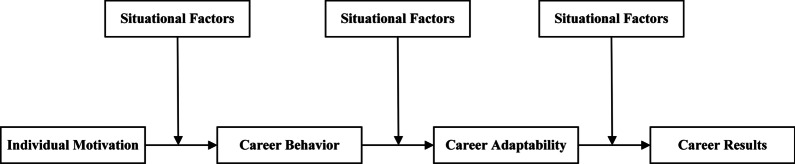
CCT Model

After the outbreak of the epidemic, the economy has been hit unprecedentedly, and the labor market is full of uncertainty. Faced with this special period, the severe employment problem of college students is also more prominent. Savickas (2002) put forward the Career Construction Theory (CCT), which holds that because the construction of college students’ career environment is uncertain, college students’ career development needs to be developed and managed by themselves [17]. The CCT model with “adaptation” as the core provides a new theme and idea for career development research from the perspective of postmodernism, in which individual characteristics, individual behavior, individual psychological state and situational factors are all important aspects that affect the results of career construction [18]. When college students are looking for a job near graduation, they will first have a preset job choice due to their own characteristics or expectations. Perfectionism is an important indicator of college students’ job expectations before they look for a job. However, the career decision-making difficulties is a result factor in finding a job. A job that meets the expectations will reduce the difficulty of choice, while too high expectations may increase the decision-making difficulty. Specifically, Fig. 1 below shows.

### Perfectionism

Perfectionism has different definitions in academics and work. Some elites regard it as an important criterion for success, which means perfectionists pursue perfection and excellence in their work. In academic work, researchers regard it as a negative personality characteristic. Since the concept was put forward, many scholars have discussed its structure, such as the division of perfectionism [19, 20] and compilation of scale [21]. In addition, many researchers have studied the relationship between perfectionism, related concepts, and personality. For example, the five-factor model of personality [16], resilience of college students [22], test anxiety [23], and career development [24, 25].

### Career adaptability

Career adaptability is an important factor for an individual to effectively cope with career uncertainty and job role ambiguity, and it refers to the individual’s coping ability when dealing with changes in current and expected tasks and career roles [13, 26]. Career adaptability plays an important role in realizing sustainable development [27]. Cultivating career adaptability can promote the transition from school to work, thus improving the chances for individuals to find high-quality jobs and helping individuals transform from university to work [28, 29]. Many scholars have measured career adaptability, including the United States, Italy, and China [30, 31]. In addition, in the relationship between career adaptability and positive psychological characteristics, the research includes the relationship between career adaptability and life satisfaction of workers with intellectual disabilities [32] and parents of children with mild intellectual disabilities [33], optimism, resilience, and life satisfaction of adolescents [34]. Regarding the relationship between career adaptability and personality, the research mainly includes self-esteem, EQ, active personality, personality characteristics, and the relationship between internal entrepreneurship and career adaptability [35–37]. In addition, some researchers have conducted follow-up research or intervention re-research on career adaptability [38, 39].

### Career decision-making difficulties

Career decision-making difficulties include indecision and indecision, which refers to the decision-making state of most people [40]. Some researchers focus on the relationship between career decision-making difficulties and personality, emotion, core self-evaluation, ambiguity management, and others [40–43]. In addition, many researchers have discussed the influencing factors of career decision-making difficulties from the career perspective, including parents’ expectations, cultural value orientation, career exploration, creative self-efficacy, social support, future time view, childhood environmental adversity, career decision-making self-efficacy, and career decision-making [26, 44].

### Perfectionism, career adaptability, and career decision-making difficulties

At present, there is insufficient research on perfectionism, career adaptability, and career decision-making difficulty, which mainly involves the research between two variables. The study of the relationship between perfectionism and career decision-making difficulties mainly includes the role of perfectionism and Big Five personalities in career indecision [45], the relationship among perfectionism, negative professional thinking, and career decision-making self-efficacy [46], and the influence of perfection ism on career pressure and career decisions [25]. In these studies, more researchers focus on the self-efficacy of career decision-making. For example, there is a positive correlation between parental support, career decision-making self-efficacy, and career adaptability of Chinese college students and Turkish high school students [47, 48]. In the study of the relationship between perfectionism and career adaptability, the main research object is college students. For example, we explored whether social support and anxiety can predict students’ perfectionism. Self-directed perfectionism positively predicts career adaptability, while social prescriptive perfectionism negatively predicts career adaptability [49].

In addition, the social expectation model [50] explains that [51]perfectionists are harsh, which makes it easy for people in career choices to achieve perfection through negative evaluation and then promotes the emergence of negative perfectionism. The Career Decision-Making Difficulty Model [51] points out that career decision-making difficulties consist of difficulties before and during career decision-making. When people who want to make ideal decisions encounter unreasonable external information and difficulties in the self-adjustment process in career decision-making, it may cause career decision-making difficulties. Therefore, we take perfectionism as an independent variable, career decision-making difficulty as a dependent variable, and career adaptability as an intermediary variable as the theoretical model of this study.

## Materials and methods

### Participants and procedure

In this study, online questionnaires were distributed to a total of 400 senior graduates from three universities in China, one in the eastern region, one in the southwestern region, and one in the central region, which can effectively eliminate the differences brought about by different regions. The study started in March 2021 to obtain relevant data and ended in April after 1 month. To avoid common methodological bias due to cross-sectional data, this study was divided into two questionnaire contents for the same set of participants, with a time interval of 6 weeks. All questionnaires were informed of the purpose of the questionnaire at the beginning. All the participants were students who voluntarily participated in the questionnaire and received a thank-you gift of 5 yuan after completing the questionnaire. A total of 392 questionnaires were collected (98.00%). Initial analysis of the returned questionnaires excluded both blank questionnaires and questionnaires with significant patterns (i.e., 111,111) that indicated sham participation. An additional 4 questionnaires were excluded when difference values between two valid test items were larger than 4 according to criteria for valid test items of the EPCD [43]. Overall, 388 questionnaires (97.00%) passed further data analysis.

### Measures

#### Multidimensional perfectionism scale

The Multidimensional Perfectionism Scale was assessed by the method of Frost et al. [52]after translation into Chinese. There were 27 items and 5 dimensions: fear of mistakes, hesitation in actions, parents’ expectation, organization, and personal standards. Among them, the organizational dimension measures individual adaptive perfectionism which means positive perfectionism in this study. To ensure the reliability of the single-dimensional variable of positive perfectionism, subsequent studies included each of its questions as a dimension in their statistics. The other four dimensions measure individual non-adaptive perfectionism belongs to negative perfectionism in this study. A five-point score is used, ranging from 1 to 5, where 1 means very inconsistent and 5 means very consistent. In this study, Cronbach’s alpha coefficient of the scale was 0.843. The negative perfectionism and positive perfectionism were 0.851 and 0.835, respectively, which have good reliability and can be used in Chinese college students.

#### College students’ career adaptability scale

The College Students’ Career Adaptability Scale was assessed by the method of Chen et al. [53]. There were 35 items and 6 dimensions: career control, career curiosity, career concern, career confidence, career adjustment, and career interpersonal relationship. A five-point score is used, ranging from 1 to 5, where 1 means very inconsistent and 5 means very consistent. In this study, Cronbach’s alpha coefficient of the scale was 0.901, and the 6-dimensional coefficient was 0.742–0.823, which can be used to evaluate the career adaptability of Chinese college students.

#### Career decision-making difficulties Scale

The Career Decision-making Difficulty Scale was assessed by the method of Tien [54] after translation into Chinese. There were 35 items and 3 dimensions: lack of preparation, difficulty in information exploration, conflict, and contradiction. A five-point score is used, ranging from 1 to 5, where 1 means very inconsistent and 5 means very consistent. In this study, Cronbach’s alpha coefficient of the scale was 0.842, and the 3-dimensional coefficient was 0.634–0.775, which can be used to evaluate the career decision-making difficulty of Chinese college students.

### Research hypothesis

**Fig. 2:**
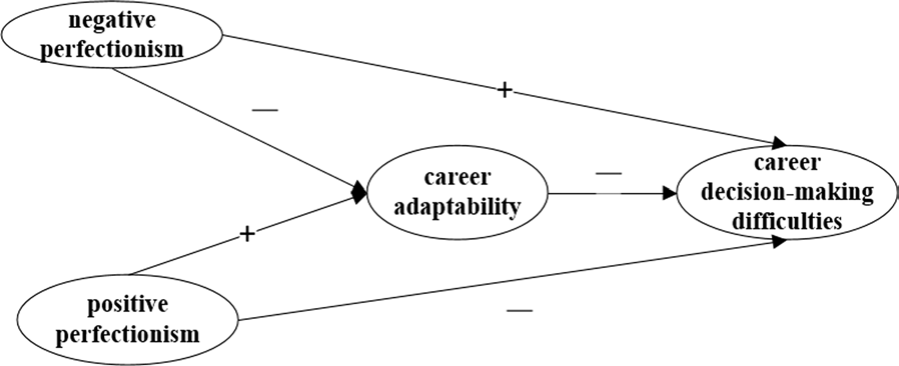
Hypothetical model of mediation

According to the analysis results of the literature review, there is a certain correlation between positive perfectionism, negative perfectionism, career adaptability, and career decision-making difficulties. Therefore, this paper proposed the following hypothesis and the model can be seen in Fig. 2.

#### **Hypothesis 1**

*The positive perfectionism of college students has a significant negative correlated with career decision-making difficulties.*

#### **Hypothesis 2**

*The negative perfectionism of college students has a significant positive correlation with career decision-making difficulties.*

#### **Hypothesis 3**

*Positive perfectionism of college students can positively predict career adaptability.*

#### **Hypothesis 4**

*Negative perfectionism of college students can negatively predict career adaptability.*

#### **Hypothesis 5**

*Career adaptability plays an intermediary role between positive perfectionism and career decision-making difficulties.*

#### **Hypothesis 6**

*Career adaptability plays an intermediary role between negative perfectionism and career decision-making difficulties.*

## Results

### Common method deviation test

Common method deviation refers to the artificial covariation among variables caused by the same data source, measurement environment, project context, and project characteristics. Common method deviation will lead to inaccurate data and misleading conclusions, so it should be controlled. Generally, common method deviation is controlled by both procedures and statistics.

Procedural control: (l) When the questionnaire is applied, the subjects are asked to answer anonymously, and the guidelines of the questionnaire and the subjects’ answers emphasize the anonymity and confidentiality of the research; (2) By choosing subjects to answer in different schools, places and periods, the measurement is separated in time and space; (3) Set a certain number of reverse scores to prevent the subjects from answering carelessly, and try to detect the true level of the subjects as much as possible.

Although the program is controlled to a certain extent, it is still difficult to eliminate common method deviation. Therefore, this study further uses the Harman single factor test to perform exploratory factor analysis on all measurement items and finds that there are 26 items with factor characteristic values greater than 1, and the maximum factor explanation variance is 15.540%, less than 40%. Therefore, it can be considered that the common method deviation is well controlled in this study, and there is no common method deviation problem.

### Descriptive statistic

In this study, perfectionism, career adaptability, and career decision-making difficulties are analyzed utilizing the mean, standard deviation, maximum value, and minimum value, among which perfectionism is divided into positive perfectionism and negative perfectionism. As shown in the following Table 1.

**Table 1:** Descriptive statistics of positive perfectionism, negative perfectionism, career adaptability, and career decision-making difficulties

Items	Min	Max	M	SD
*Positive perfectionism (organization)*	1.21	5.00	3.75	0.74
*Negative perfectionism*	1.53	5.00	2.83	0.58
fear of mistakes	1.00	5.00	2.31	0.61
parents’ expectation	1.00	5.00	2.74	0.81
Hesitation in actions	1.00	5.00	3.12	0.75
Personal standards	1.00	5.00	3.21	0.78
*Career adaptability*	1.62	4.95	3.81	0.52
Career control	1.80	5.00	3.81	0.49
Career curiosity	1.23	5.00	3.73	0.64
Career concern	1.64	5.00	3.48	0.65
Career confidence	1.33	5.00	3.79	0.57
Career adjustment	1.31	5.00	3.71	0.58
Career interpersonal relationship	1.20	5.00	3.12	0.65
*Career decision-making difficulties*	1.33	4.28	2.71	0.57
Lack of preparation	1.00	4.87	2.59	0.64
Difficulty in information exploration	1.00	4.96	2.72	0.71
Conflict and contradiction	1.00	4.77	2.76	0.57

In terms of perfectionism. The average value of positive perfectionism is significantly higher than the median value, which indicates that the level of positive perfectionism of college students is at a high level. The total score of negative perfectionism and the scores of fear of mistakes and parents’ expectations are lower than the median, but the scores of personal standards and hesitation in actions are higher than the median. After entering the university, college students have more initiative and autonomy in their study and life and pursue organization and order in doing things. At the same time, due to further separation from parental control, the influence of parental expectations on college students gradually decreases, which leads students to have high self-confidence and will not deny themselves because of a temporary failure. However, college students cannot set their own personal standards according to the actual situation. Moreover, due to their lack of ability and the inability to see a longer-term future, they cannot predict the mistakes and failures they will face, and they will have doubts when they act.

In terms of career adaptability. The scores of career adaptability in general and all dimensions are higher than the median, which indicates that college students’ career adaptability is at a high level. Currently, universities no longer only emphasize grades, which makes college students have more extracurricular activities after studying to get on well with classmates and cultivate their own interests and hobbies. In addition, the popularity of the Internet also enables college students to understand the latest information in all aspects faster, which is conducive to enhancing their self-confidence and promoting their good interpersonal relationships and ultimately improving their career adaptability. However, the score of the career control dimension is low. The reason is that most college students learn books mechanically and have little or no chance to do social practice related to their own majors. When they need to do something, they will feel at a loss, anxious, and uncontrollable.

In terms of career decision-making difficulties. This study found that the average values of career decision-making difficulties in general and all dimensions are slightly lower than the median, which indicates that contemporary college students have some degree of career decision-making difficulties. Specific performance can be classified into three points: lack of awareness and relevant knowledge of career planning, inability to prepare for career planning in advance, and always think that career choice should be considered by college students who are nearing graduation.

### Correlation analysis

In this study, the Pearson correlation coefficient was used to test the relationship among positive perfectionism, negative perfectionism, career adaptability, and career decision-making difficulties [55]. The specific results are shown in Table 2 below.

**Table 2:** Correlation analysis

	1	2	3	4
1. Positive perfectionism	–			
2. Negative perfectionism	0.185**	–		
3. Career adaptability	0.403**	−0.084*	–	
4. Career decision-making difficulties	−0.139**	0.315**	−0.502**	–

*:*p* < 0.05; **:*p* < 0.01

The relationship between the four variables shows a significant pairwise correlation. Positive perfectionism is positively correlated with career adaptability (r = 0.403; *p* < 0.01) and negatively correlated with career decision-making difficulties (r=−0.139; *p* < 0.01). Negative perfectionism was negatively correlated with career adaptability (r=−0.084; *p* < 0.05) and positively correlated with career decision-making difficulty (r = 0.315; *p* < 0.01). There is a significant negative correlation between career adaptability and career decision-making difficulty (r=−0.502; *p* < 0.01). Thus, the research hypotheses 1–4 were confirmed.

### Regression analysis of perfectionism and career decision-making difficulties

**Fig. 3:**
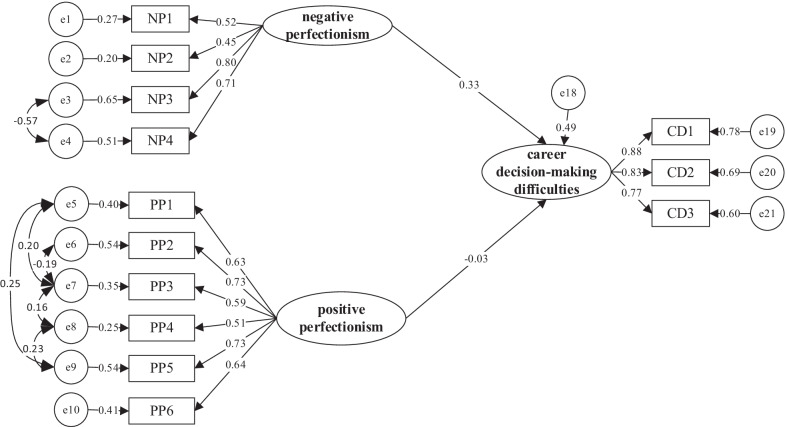
Regression model. *Note*: NP: dimensions of negative perfectionism; PP: questions of positive perfectionism; CD: dimensions of career decision-making difficulties

Based on the correlation analysis of positive perfectionism, negative perfectionism, career adaptability, and career decision-making difficulties, this paper constructs a regression model of positive perfectionism, negative perfectionism, and career decision-making difficulties combined with theoretical analysis and investigates the predictive effects of positive perfectionism and negative perfectionism on the career decision-making difficulties of college students. The results show that X^2^/df = 3.186, RMSEA = 0.075, NFI = 0.952, IFI = 0.964, CFI = 0.951, GFI = 0.973, and AGFI = 0.969, which indicates that this model has a good fitting degree. The specific results are shown in Fig. 3 below.

In addition, the main path coefficients of the regression model show that the standardized regression coefficient of positive perfectionism to career decision-making difficulties is −0.171, *p* < 0.01. The standardized regression coefficient of negative perfectionism to career decision-making difficulties is 0.479, *p* < 0.01. The regression path coefficient differences are extremely significant, which shows that positive perfectionism can negatively predict career decision-making difficulties, and college students with higher positive perfectionism can reduce the occurrence of career decision-making difficulties. Negative perfectionism can positively predict career decision-making difficulties, while higher negative perfectionism of college students is not conducive to the smooth production of career decision-making.

### Mediating effect analysis

**Fig. 4:**
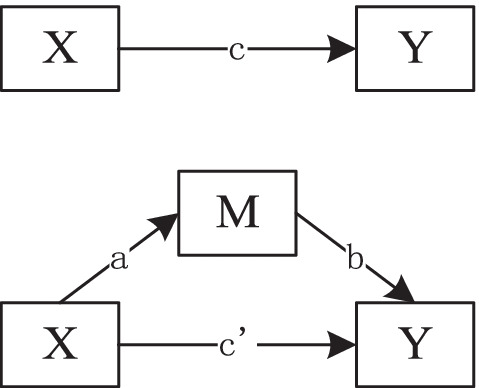
Schematic diagram of a mediation model

According to Hayes and Scharkow, mediation analysis can effectively analyze the influence of variable X on variable Y, which makes research progress in statistical methods and obtains more in-depth research results [56]. In addition, if the independent variable x has a certain influence on the dependent variable y through the variable M, then the variable m is the intermediary variable of X and Y, and the specific mediating effect can be described by the regression equation path diagram in Fig. 4.

Based on the above model, this study uses Amos 22.0 software to test the mediating effect of career adaptability on perfectionism and career decision-making difficulties. The results show that X^2^/df = 2.564, RMSEA = 0.069, NFI = 0.948, IFI = 0.959, CFI = 0.963, GFI = 0.968, and AGFI = 0.971, which indicates that this model has a good fitting degree.

**Table 3:** Main path coefficients of the mediation model

Path	Estimate	SE	CR	*P*
NP → CA	−0.711	0.054	−3.174	0.001
PP → CA	0.298	0.048	6.664	0.000
CA → CDMD	−0.589	0.087	−6.989	0.000
NP → CDMD	0.331	0.059	5.411	0.000
PP → CDMD	−0.026	0.046	−0.608	0.243

Furthermore, this study analyzes the main path coefficients of the mediation model of perfectionism, career adaptability, and career decision-making difficulties of college students, and the results are shown in Table 3.

**Fig. 5:**
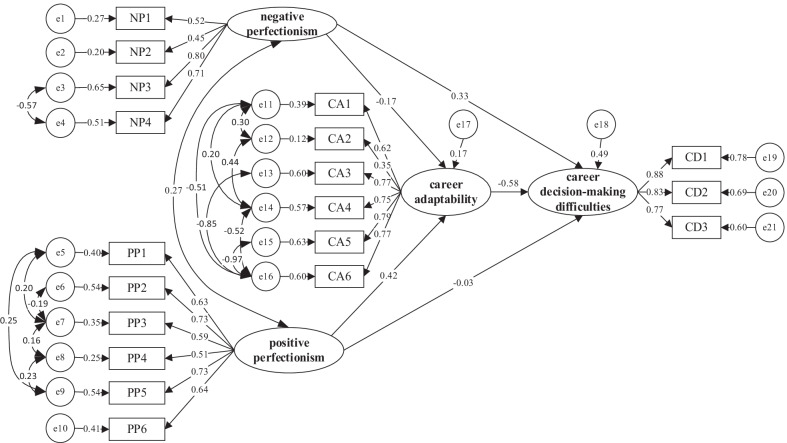
Mediation model. *Note*: NP: dimensions of negative perfectionism; PP: questions of positive perfectionism; CA: dimensions of career adaptability; CD: dimensions of career decision-making difficulties

From the above data, it can be seen that the fitting index of this model, except for the influence of positive perfectionism on career decision-making difficulties, does not meet the standard, and other fitting indexes all meet the standard, so the model fits well. It shows that career adaptability plays an intermediary role in the role of positive perfectionism and negative perfectionism in career decision-making difficulties. Specifically, career adaptability plays a partial mediating role in the relationship between negative perfectionism and career decision-making difficulties. It plays a complete intermediary role in the relationship between positive perfectionism and career decision-making difficulties, and the research hypothesis holds. Therefore, this study establishes a mediation model, as shown in Fig. 5.

Negative perfectionism directly affects the career decision-making level of college students; the higher the score of negative perfectionism of college students is, the easier it is to make career decision-making difficulties. Negative perfectionism can also influence career decision-making difficulties through career adaptability; if college students have higher negative perfectionism, it will be detrimental to the development of career adaptability, and college students with lower career adaptability are more likely to have career decision-making difficulties.

Positive perfectionism does not directly affect career decision-making difficulties but exerts an influence on career decision-making difficulties completely through career adaptability. College students’ higher positive perfectionism is conducive to the development of their career adaptability, and the improvement of career adaptability helps college students make career decisions smoothly, resulting in fewer career decision-making difficulties. Thus, the research hypotheses 5 and research hypotheses 6 were confirmed.

After fitting the mediation analysis model, it is necessary to decompose the mediation effect. Usually, the contents of decomposition mainly include (1) the size of the mediation effect; (2) the ratio of the intermediary effect to the total effect, namely, ab/(ab + c’); (3) the ratio of the intermediary effect to the direct effect, namely, ab/c’; and (4) the size of the specific mediating effect, that is, the total mediating effect through a certain mediating variable.

As shown in Tables 4, in the mediation model of negative perfectionism, career adaptability, and career decision-making difficulties, the direct effect is 0.334, the mediating effect is 0.099, and the total effect is 0.433. In the mediation model, ab and c’ have the same sign and are significant, which indicates that the mediation is part of the mediation, and the mediation effect accounts for 22.9% of the total effect.

**Table 4:** Effect analysis of negative perfectionism

Effect	Effect value	Result
Direct effect	c’	0.334
Mediating effect	ab	0.099
Total effect	ab + c’	0.433
The ratio of mediating effect to total effect	ab/(ab + c’)	0.229
The ratio of mediating effect to direct effect	ab/ c’	0.296

As shown in Tables 5, in the mediation model of positive perfectionism, career adaptability, and career decision-making difficulties, the direct effect is −0.032, the mediation effect is −0.244, and the total effect is −0.276. In the mediation model, ab and c’ have the same sign, but c’ is not significant, which indicates that the mediation model is complete mediation, and the mediation effect accounts for 88.4% of the total effect.

**Table 5:** Effect analysis of positive perfectionism

Effect	Effect value	Result
Direct effect	c’	−0.032
Mediating effect	ab	−0.244
Total effect	ab + c’	−0.276
The ratio of mediating effect to total effect	ab/(ab + c’)	0.884
The ratio of mediating effect to direct effect	ab/ c’	7.625

## Discussion

### College students pursue closer perfection

By analyzing the overall situation of positive and negative perfectionism, it is found that the score of positive perfectionism is higher than the median, while the score of negative perfectionism is opposite, which indicates that the level of positive perfectionism is higher than that of negative perfectionism.

Firstly, the measurement of positive perfectionism shows that college students score higher, which shows that college students pay attention to the organization in their daily study and life, have a stronger motivation to pursue success, and can set practical goals for themselves. At the same time, they have positive self-awareness, and their emotional performance is more stable in the face of failure. Secondly, the measurement of negative perfectionism shows that the negative perfectionism of college students is also remarkable, which shows that their character is unstable and that they are afraid of failure when they pursue success. In addition, there is a situation of setting unrealistic or even unreachable goals, which cannot be attributed reasonably when faced with failure, and then the reason for failure can be attributed to the lack of self-ability. This may also lead to the excessive pursuit of detail and order in the implementation of the plan, and it is easier to hesitate when faced with decision-making. Finally, college students tend to set higher personal standards and goals for themselves and be strict with themselves. However, due to the lack of persistence in the implementation process, they can only see the current development of things but not the future development direction of things and the difficulties they will face. They cannot see danger in the future and will not worry about mistakes, which will lead to hesitation and doubt in the process of doing things.

### Career adaptability is unbalanced, and the control is poor

The results of the analysis indicate that college students’ career adaptability is at a good level, as their scores exceed the median. The scores of career concern and career curiosity are high, which shows that college students pay attention to the occupation they are interested in consciously and the establishment of the interpersonal relationship after studying. It also helps them to have strong self-confidence in entering society after graduation and prepare for tasks and problems in different stages of career development. However, this degree of preparation is not enough to support them in solving their career problems smoothly.

From all dimensions, the development level of college students’ career adaptability is unbalanced, and there are obvious deficiencies. Owing to lacking autonomy, college students cannot make career decisions independently and have low career control ability. At the same time, they cannot position themselves accurately, which made them lack understanding of the environment and curiosity about the profession. This conclusion has also been confirmed that many college students are not interested in their major and cannot accurately understand the relationship between their major and their future occupation [57]. Due to the lack of career planning guidance, college students cannot clearly plan their career development direction and lack their career goals. Therefore, the development level of career adaptability of college students still needs to be improved. For example, career coaching program can better help students seriously and carefully explore and treat their career and improve their career readiness [58].

### Low career decision-making ability leads to career decision-making difficulties

The analysis of college students’ career decision-making difficulties shows that college students have a moderate degree of career decision-making difficulties. In the three dimensions, the scores from low to high are lack of preparation, difficulty in information exploration, and information conflict. College students have the highest scores of career decision-making difficulties due to information conflicts. With the acceleration of information updates, the credibility of public-oriented information is greatly reduced. It is difficult for college students who have not officially entered society to distinguish between true and false information. To prevent being deceived, college students often hesitate to make career decisions. In addition, many college students do not consider work until they graduate, and they have many unreasonable beliefs, such as “staying in a big city” and “going into a big company”. These unreasonable ideas mislead their career choices and make them hesitant and miss good opportunities in the decision-making process. Finally, important people such as the “parents” of college students in daily life will influence their career choices. These factors will lead to contradictions and conflicts in the career decision-making process of college students.

### Positive perfectionism and negative perfectionism have opposite effects on career adaptability and career decision-making difficulties

This study shows that positive perfectionism is positively correlated with career adaptability and negatively correlated with career decision-making difficulties. Negative perfectionism is negatively correlated with career adaptability and positively correlated with career decision-making difficulties. There is a significant negative correlation between career adaptability and career decision-making difficulties. Positive perfectionism has a positive predictive effect on career adaptability, while negative perfectionism has a negative predictive effect on career adaptability, in which career adaptability plays an intermediary role. This basically confirms the initial hypothesis of this study.

First, perfectionists want to make career decisions that they think are perfect and satisfy themselves and other important people around them. Among them, negative perfectionists are afraid of failure and pursue unrealistic “perfection” too much. In addition, they will experience anxiety and fear of failure in the process of pursuing, thus aggravating the difficulty of career decision-making. Positive perfectionists set practical goals for themselves. They can collect relevant professional information and reasonably analyze the pros and cons while pursuing success to make career decisions smoothly.

Second, the behavioral motivation of negative perfectionists is to avoid failure, and they will set unrealistic or unreachable goals to avoid blaming their own lack of ability when they fail, so negative perfectionism is not conducive to the cultivation of career adaptability. Active perfectionists’ behavioral motivation is to pursue success. They will set practical goals and be accompanied by positive self-evaluation. At the same time, they believe that success can happen at any time. Therefore, an active perfectionist personality is conducive to an increase in individual career adaptability.

Finally, individuals with high career adaptability pay more attention to the occupations they are interested in and can actively collect relevant information about occupations. In the process of work, they can flexibly cope with all kinds of unexpected situations and adapt to the change of work more quickly, so they can make career decisions more easily.

Career adaptability plays an intermediary role in the relationship between positive perfectionism and negative perfectionism on career decision-making difficulties. Specifically, career adaptability plays a completely mediating role in the relationship between positive perfectionism and career decision-making difficulties. Positive perfectionism affects career decision-making difficulties completely through career adaptability; that is, college students with higher positive perfectionism can promote the development of their career adaptability. An increase in career adaptability will reduce the difficulty of college students’ career decisions. In addition, career adaptability plays a mediating role in the relationship between negative perfectionism and career decision-making difficulties; that is, negative perfectionism can directly lead to career decision-making difficulties and can also exert influence on career decision-making difficulties through career adaptability. If college students have higher negative perfectionism, it is not conducive to the development of their own career adaptability, while low career adaptability will make individuals have more career decision-making difficulties.

## Conclusions

The results of this study show that college students will more or less pursue “perfect”, but often with their own preferences to pursue, can not be done based on the rational analysis of things to pursue perfect, resulting in college students doing things that lack of persistence, the future planning is not clear. Such a status quo makes college students in the learning process of their work after graduation employment problems consider less, not enough comprehensive consideration, and ultimately unable to make career decisions.

At the same time, after descriptive statistics, correlation analysis, and intermediary effect analysis of positive perfectionism, negative perfectionism, career adaptability, and career decision-making difficulties of college students, it is found that on the whole, college students have higher positive perfectionism and negative perfectionism, and their career adaptability is also at a higher level, but there is a higher degree of career decision-making difficulties. From the analysis of the mediating effect, positive perfectionism of college students can reduce the difficulty of career decision-making, and career adaptability plays a completely mediating role. Negative perfectionism of college students will lead to difficulties in career decision-making, in which career adaptability plays a mediating role.

## Limitations

In summary, despite the significant findings obtained in this study, there are still some limitations in this study. First, the subjects were selected from three different regions in China, but due to the limited sample size, this study did not obtain significant differences in the results because of the regional differences as other studies. Second, because this study used a questionnaire format to collect data, although multiple formats were used to ensure possible common methodological bias from self-reporting, it is inevitable that the results are prone to change due to individual growth. Therefore, future studies could capture the differences in the variables included in this study due to individual growth cycles on a follow-up survey. Third, deficiencies in the study hypothesis model. This study proposed a research hypothesis on the relationship between the three variables based on the existing literature review and research deficiencies, but more existing research findings may be missed due to the researcher’s reading limitations, which is the key for future studies to be considered in depth.

## Data Availability

The data that support the findings of this study are available from the corresponding author. Restrictions apply to the availability of these data, which were used under licence for this study. Data are available from the authors with the permission of Nanjing Normal University.

## Abbreviations

NP: Negative perfectionism
PP: Positive perfectionism
CA: Career adaptability
CDMD: Career decision-making difficulties
